# Inhibition of NF-*κ*B prevents the acidic bile-induced oncogenic mRNA phenotype, in human hypopharyngeal cells

**DOI:** 10.18632/oncotarget.23143

**Published:** 2017-12-12

**Authors:** Dimitra P. Vageli, Sotirios G. Doukas, Clarence T. Sasaki

**Affiliations:** ^1^ The Yale Larynx Laboratory, Department of Surgery, Yale School of Medicine, New Haven, CT, USA

**Keywords:** bile acids, NF-kB inhibitor, hypopharyngeal carcinogenesis, mRNA phenotype, laryngopharyngeal reflux

## Abstract

Bile-containing gastro-duodenal reflux has been clinically considered an independent risk factor in hypopharyngeal carcinogenesis. We recently showed that the chronic effect of acidic bile, at pH 4.0, selectively induces NF-*κ*B activation and accelerates the transcriptional levels of genes, linked to head and neck cancer, in normal hypopharyngeal epithelial cells. Here, we hypothesize that NF-*κ*B inhibition is capable of preventing the acidic bile-induced and cancer-related mRNA phenotype, in treated normal human hypopharyngeal cells. In this setting we used BAY 11-7082, a specific and well documented pharmacologic inhibitor of NF-*κ*B, and we observed that BAY 11-7082 effectively inhibits the acidic bile-induced gene expression profiling of the NF-*κ*B signaling pathway (down-regulation of 72 out of 84 analyzed genes). NF-*κ*B inhibition significantly prevents the acidic bile-induced transcriptional activation of NF-*κ*B transcriptional factors, RELA (p65) and c-REL, as well as genes related to and commonly found in established HNSCC cell lines. These include anti-apoptotic bcl-2, oncogenic STAT3, EGFR, ∆Np63, TNF-α and WNT5A, as well as cytokines IL-1β and IL-6. Our findings are consistent with our hypothesis demonstrating that NF-*κ*B inhibition effectively prevents the acidic bile-induced cancer-related mRNA phenotype in normal human hypopharyngeal epithelial cells supporting an understanding that NF-*κ*B may be a critical link between acidic bile and early preneoplastic events in this setting.

## INTRODUCTION

Many forms of Head and neck squamous cell carcinoma (HNSCC) have been attributed to known risk factors such as tobacco smoking and alcohol consumption [1–3], whereas extra-esophageal or laryngopharyngeal reflux disease (ERD or LPR) is now also considered to be an independent risk factor in laryngopharyngeal carcinogenesis [4–6]. Although the clinical prevalence and magnitude of gastroduodenal reflux is not fully known, there seems to be growing evidence that approximately 50% to 86% of patients with gastro-esophageal reflux disease (GERD) present with mixed gastric and duodenal fluids (bile acids) in their refluxates [7–9]. According to Covington et al bile-containing enterogastric reflux is much more common than previously appreciated [10]. Gali et al. showed that during LPR gastroduodenal fluid (GDF) reaches the epithelium of the upper aero-digestive tract and contributes to the development of inflammatory and neoplastic events [6]. The precise role of bile-containing GDF in hypopharyngeal cancer and the underlying mechanism of its carcinogenic effect remain unclear. In the initiation and progression of HNSCC, several oncogenic pathways have been identified. These commonly include EGFR/Ras/RAF/MAPK, Akt/PI3K/mTOR, ΙΚΚ/NF-κB, STAT3, and wnt/β-catenin [11–20]. In this setting, our recent *in vitro* and *in vivo* explorations demonstrate that extra-esophageal reflux may play a role in laryngopharyngeal carcinogenesis, mediated by NF-κB [21–23]. Recently published data demonstrate that bile, particularly at pH ≤ 4.0 is capable of inducing NF-κB activation and oncogenic mRNA phenotype [21, 22]. Specifically, bile at molar concentrations previously reported in symptomatic GERD patients [8] at pH ≤ 4.0 can induce significant activation of NF-κB and related genes associated with oncogenic function, in both cultured normal hypopharyngeal keratinocytes and premalignant lesions of exposed murine laryngopharyngeal mucosa [22]. According to Ulualp et al. studies of 24-hour ambulatory pH monitoring in the pharynx of patients, a drop below pH 4.0 is not uncommon and is considered diagnostic of a reflux event [24], suggesting that acid may contribute to GDF-induced inflammatory and neoplastic events. Moreover, Dvorak et al. showed that bile at acidic pH may potentially induce DNA damage [25]. Here we hypothesize that NF-κB inhibition is capable of preventing the acidic bile-induced and cancer-related mRNA phenotype in treated normal hypopharyngeal cells, *in vitro*, further emphasizing the understanding that NF-κB is a critical link between acidic bile and preneoplastic events.

Nuclear factor-kappB is widely considered a key mediator between chronic inflammation and cancer. Many studies underscore the understanding that NF-κB regulates the transcriptional activation of genes with anti-apoptotic function, cell proliferation, tumor initiation and progression, as well as genes mediating epithelial mesenchymal transition (EMT) [11–13]. Lee at al suggest that the constitutive activation of NF-κB provides an alternative mechanism for head and neck carcinogenesis [26]. Specifically, they show that there are subtypes of head and neck cancer that demonstrate constitutive activation of NF-κB with significant transcriptional alterations in clusters of NF-κB target genes.

The canonical pathway of NF-κB activation includes phosphorylation of IκB-α, that leads to nuclear translocation of heterodimers p50/Rela or p50/cRel, and consequent binding to the promoters of target genes and regulating their expression. NF-κB activation induces a signaling pathway by accelerating the expression of several receptors, ligands and transcriptional factors that regulate NF-κB activation and immune response through NF-κB, while inhibition of NF-κB negatively affects the gene expression profiling of the NF-κB signaling pathway, and has been considered promising for improving anti-cancer therapies [27]. The inhibition of NF-κB through BAY 11-7082 [(E)-3-(4-methylphenylsulphonyl)-1-propenenitrile], has been well documented by others by inhibiting IκB-α phosphorylation, blocking proteosomal degradation of IκB-α and allowing NF-κB to sequester in the cytoplasm in an inactivated state [28–30]. Selective inhibition of NF-κB and its related transcriptional phenotypes may clarify the role of NF-κB as a critical component linking acidic bile to preneoplastic events.

## RESULTS

### BAY 11-7082 inhibits the acidic bile-induced NF-κB activation and bcl-2 cytoplasmic accumulation in normal human hypopharyngeal cells

**Figure 1:**
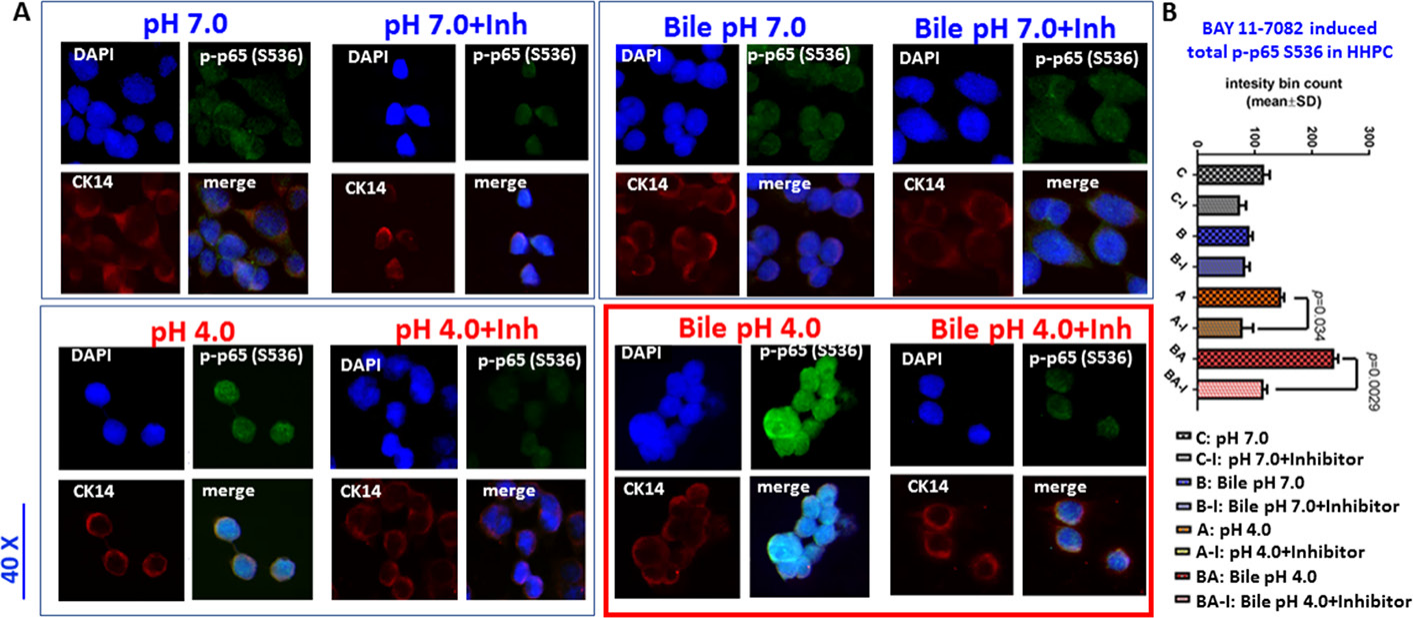
BAY 11-7082 inhibits the acidic bile-induced nuclear translocation of NF-κB (p65) phosphorylated at Ser536 in human primary hypopharyngeal cells Immunofluorescence staining for phospho-NF-κB (p-p65 S536) reveals that application of BAY 11-7082 (**A**) decreases the nuclear translocation of p-p65 in acidic bile treated human hypopharyngeal primary cells (HHPC), demonstrating decreased p-p65 nuclear levels (green: p-p65 S536; red: CK14 for cytoplasmic staining; blue: DAPI for nuclear staining), and (**B**) significantly decreases total p-p65 (Ser536) levels, particularly in acid (pH 4.0) and acidic bile (pH 4.0) groups (*p* values by *t*-test; multiple comparisons by Holm-Sidak; GraphPad Prism 6.0).

We observed that the nuclear localization of phospho-NF-κB (p-p65 S556) was inhibited by BAY 11-7082, in human hypopharyngeal primary cells (HHPC) exposed to acidic bile at pH 4.0. This observation was characterized by decreased p-p65 nuclear staining by using an immunofluorescence (IF) assay (Figure 1). We also found that BAY 11-7082 inhibited p-p65 nuclear localization in HHPC exposed to acid alone (pH 4.0). This observation was also characterized by decreased p-p65 nuclear staining, implying that NF-κB inhibitor blocks acid-induced p-p65 translocation to the nucleus. However, we observed that BAY 11-7082 induced minimal changes in nuclear staining of p-p65 in HHPC exposed to bile at pH 7.0 or to neutral control (Figure 1), compared to HHPC at pH 4.0. Finally, we showed that cells exposed to DMSO, exhibited patterns of weak nuclear p-p65 staining like that of the untreated control, implying that the solubilizing vehicle for BAY 11-7082 had no effect on p-p65 localization or expression (see Supplementary Figure 1 online).

Staining with CK14 supports the observation that a proportion of p-p65 remains in the cytoplasmic compartment of HHPC treated by bile at pH 7.0 with inhibitor. In contrast, NF-κB inhibitor seems to reduce both cytoplasmic and nuclear p-p65 levels in HHPC treated by bile at pH 4.0, implying reduced protein expression in this group.

Statistical analysis revealed that BAY 11-7082 effectively reduced total p-p65 (Ser536) levels in treated HHPC, with significant difference in those exposed to acid alone (pH 4.0) (*p* = 0.037), and particularly to acidic bile (pH 4.0) (*p* = 0.0026), supporting the contribution of acid (pH 4.0) in NF-κB activation of treated cells (Figure 1B) (*t*-test; means ± SD; multiple comparisons by Holm-Sidak). These data demonstrate that acidic bile-related NF-κB activation and NF-κB expression are substantially reduced by BAY 11-7082, in treated HHPC.

**Figure 2:**
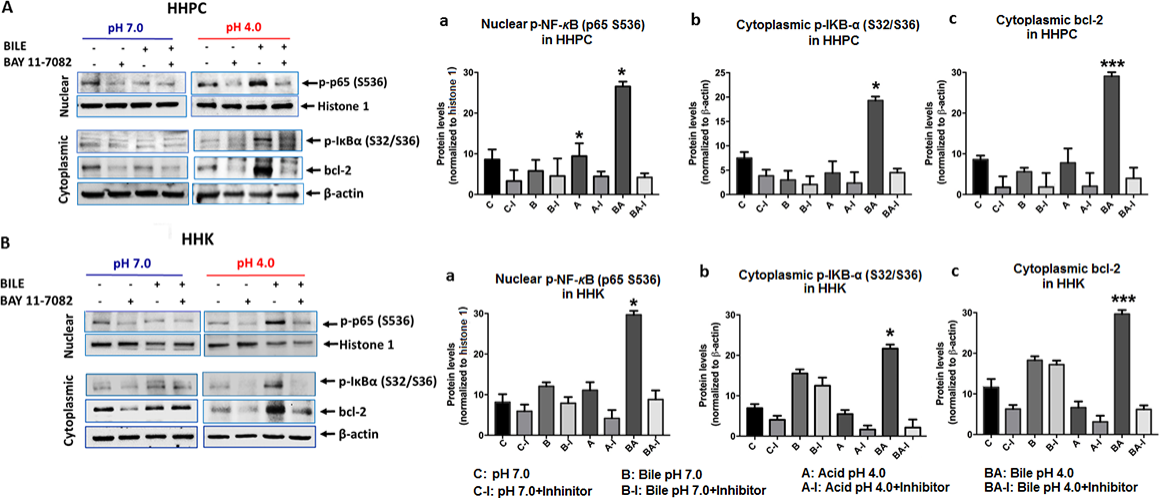
ΒΑΥ 11-7082 inhibits the acidic bile-induced NF-*κ*B activation and bcl-2 expression in normal human hypopharyngeal cells Western blot analysis was performed in nuclear and cytoplasmic protein extracts of treated (**A**) human hypopharyngeal primary cells (HHPC) and (**B**) human hypopharyngeal keratinocytes (HHK) (with and without BAY 11-7082) for (a) p-NF-*κ*B (p65 S536), (b) p-IκB-α (Ser32/36) and (c) bcl-2 (ONE-WAY ANOVA; Kruskal-Wallis, ^*^p < 0.05; ^**^p < 0.005; ^***^p < 0.0005 GraphPad Prism 6.0) (Nuclear p-NF-κB protein levels were normalized to Histone 1; cytoplasmic p-IκB-α and bcl-2 protein levels were normalized to β-actin. Data of three independent assays).

To further analyze the effect of BAY 11-7082 on NF-κB activation and bcl-2 expression, we performed a western blot analysis for phospho-NF-κB (p65 S356) (∼65 kDa), phospho-inhibitor kappaB-a (p-IκB-α S32/S36) (∼40 kDa) and bcl-2 (∼28 kDa), using Histone 1 (∼30 kDa) and β-actin (∼37 kDa) for normalization of the expression, in nuclear and cytoplasmic protein fractions of the treated cells (Figure 2). (i) We observed an inhibition of NF-κB activation and bcl-2 cytoplasmic accumulation in both HHPC and HHK exposed to bile at pH 4.0 with BAY 11-7082, compared to cells exposed to bile at pH 4.0 without BAY 11-7082. This inhibition was evidenced by significantly decreased phospho-NF-κB nuclear levels in cells exposed to bile at pH 4.0 (Figure 2A-a, 2B-a), accompanied by reduced cytoplasmic p-IκB-α levels (Figure 2A-b, 2B-b) and cytoplasmic bcl-2 ratios (Figure 2A-c, 2B-c) (*p* < 0.05; by paired *t*-test; Graph Pad Prism 6.0). (ii) We also found a decrease in NF-κB activation in HHPC exposed to acid (pH 4.0) plus BAY 11-7082, compared to those exposed to acid alone, without NF-κB inhibitor, demonstrating that NF-κB inhibitor suppressed NF-κB activation induced by low pH (Figure 2A-a, 2B-a). This event was evidenced by a decrease of cytoplasmic p-IκB-α levels in cells exposed to acid alone (pH 4.0) (Figure 2A-b, 2B-b). (iii) On the other hand, we found minimal changes of nuclear NF-κB (Figure 2A-a, 2B-a) and cytoplasmic p-IκB-α (Figure 2A-b, 2B-b) levels in both HHPC and HHK treated with bile at pH 7.0 plus BAY 11-7082, compared to those exposed to bile at pH 7.0 without BAY 11-7082, again supporting our observations by IF assay.

**Figure 3:**
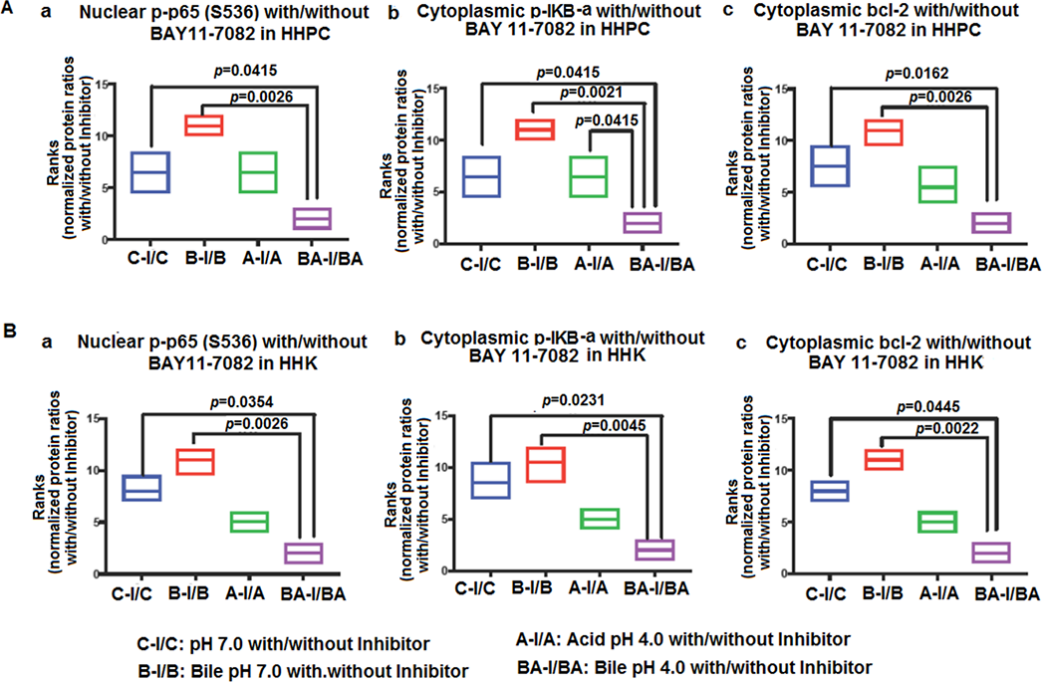
Acidic bile (pH 4.0) treated normal human hypopharyngeal cells demonstrate the most significant reduction of activated NF-*κ*B, p-IκB-α and bcl-2 protein levels in the presence of ΒΑΥ 11-7082 (**A**) Human hypopharyngeal primary cells (HHPC), and (**B**) Human hypopharyngeal keratinocytes (HHK). Graphs created by Graph Pad Prism 6.0 software reveal ranks of (a) nuclear p-NF-κB(p65 S536), (b) cytoplasmic p-IκB-α (Ser32/36) and (c) cytoplasmic bcl-2 normalized protein levels (with/without NF-κB inhibitor) between different experimental and control groups, in treated HHPC and HHK, by western blot analysis. (ONE-WAY ANOVA; Kruskal-Wallis; GraphPad Prism 6.0). (Nuclear p-NF-κB protein levels were normalized to Histone 1; cytoplasmic p-IκB-α and bcl-2 protein levels were normalized to β-actin. Data of of three independent experiments).

Further, we observed that HHPC and HHK exposed to bile plus inhibitor at pH 4.0 demonstrated the lowest relative expression ratios (with/without inhibitor) of activated NF-κB (Figure 3A-a, 3B-a), cytoplasmic p-IκB-α levels (Figure 3A-b. 3B-b), and bcl-2 (Figure 3A-c, 3B-c), with a significant difference compared to neutral control (pH 7.0), neutral bile (pH 7.0) or acid alone (pH 4.0) (*p* < 0.05; ONE WAY ANOVA, Kruskal-Wallis, GraphPad 6.0).

Taken together, BAY11-7082 effectively prevented the bile-induced activation of NF-κB at pH 4.0. Moreover, we showed that the effect of BAY 11-7082 resulted in a significant reduction of bile-induced cytoplasmic bcl-2 accumulation, particularly at acidic pH. NF-κB inhibition was significantly more effective in preventing NF-κB activation and bcl-2 expression in cells repetitively exposed to bile at pH 4.0, compared to bile at pH 7.0 or acid alone (pH 4.0).

### BAY 11-7082 inhibits the acidic bile-induced NF-κB transcriptional activity at pH 4.0 in treated normal human hypopharyngeal cells

**Figure 4:**
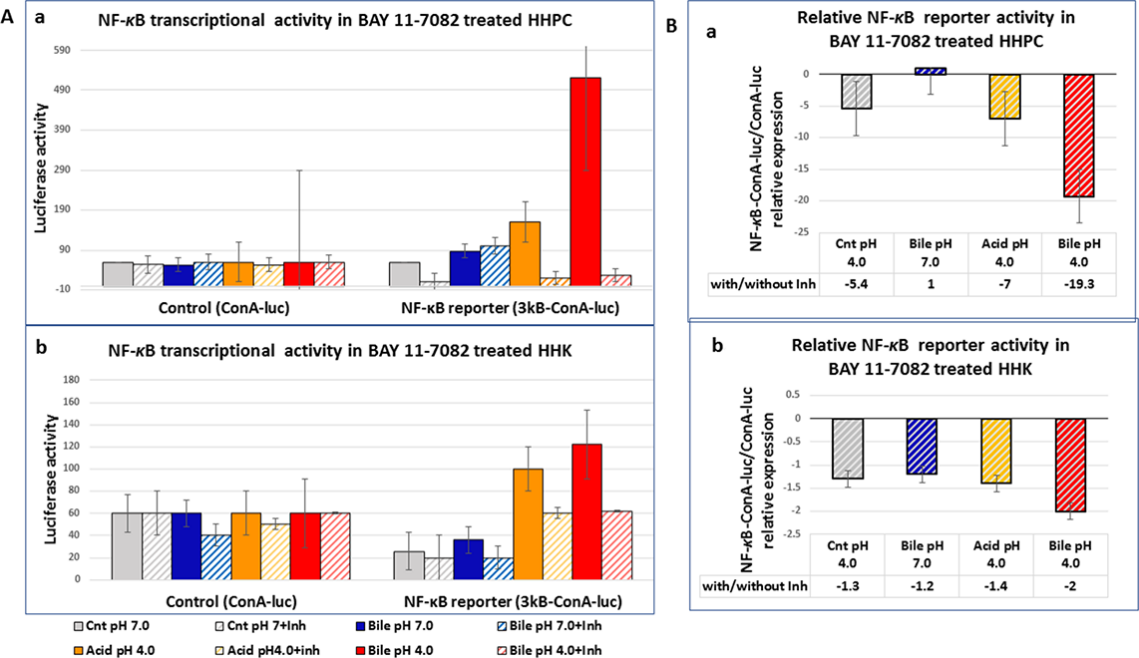
BAY 11-7082 suppresses NF-*κ*B transcriptional activity in HHPC and HHK treated by acidic bile (**A**) Graphs depict the luciferase activity (mean±SD of two independent experiments) in (a) HHPC and (b) in HHK transfected with control (conA-luc) or NF-κB responsive (3kB-conA-luc) luciferase reporter. (**B**) Graphs depict the NF-κB relative (3kB-conA-luc/conA-luc) transcriptional activity levels, in (a) HHPC and (b) HHK, showing that BAY 11-7082 suppresses NF-κB transcriptional activity in treated cells, particularly in the acidic bile-treated groups.

Luciferase assay revealed lower NF-κB transcriptional activity in both HHPC and HHK exposed to BAY 11-7082, compared to those cells treated without NF-κB inhibitor, as shown by the negative ratios of relative NF-κB activity in Figure 4. HHK and particularly HHPC (Figure 4A-a,b), exposed to bile at pH 4.0 plus BAY-11-7082, exhibited the most intense inhibition of NF-κB transcriptional activity, compared to other experimental media or controls (Figure 4A, 4B). This finding was evidenced by the lowest negative ratios of relative NF-κB activity (with/without BAY 11-7082) (Figure 4A, 4B). In contrast, luciferase assay revealed that both HHPC and HHK exposed to bile at pH 7.0 plus BAY-11-7082 demonstrated minor changes of NF-κB transcriptional activity compared to cells exposed to bile at pH 7.0 without NF-κB inhibitor. This finding was represented by the highest negative ratios of relative NF-κB activity in HHPC and HHK exposed to bile at pH 7.0.

### BAY 11-7082 prevents the acidic bile-induced transcriptional levels of NF-κB and related genes with anti-apoptotic or oncogenic function in treated normal human hypopharyngeal cells

**Figure 5:**
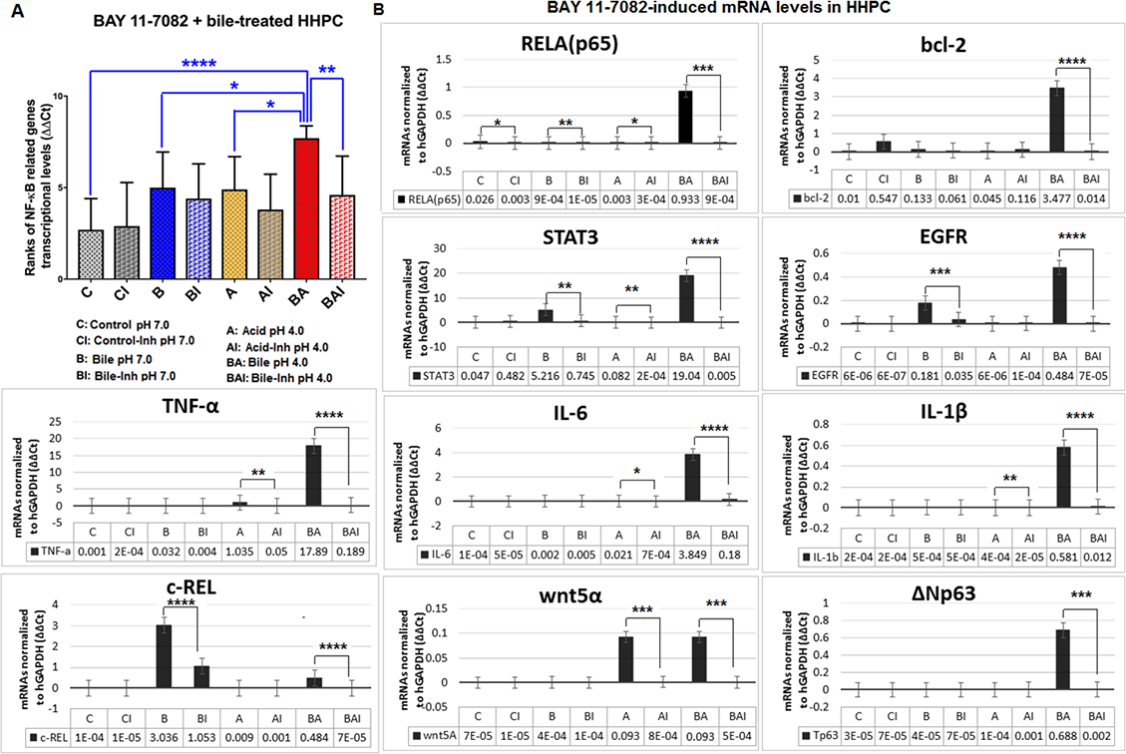
Inhibition of NF-*κ*B prevents the acidic bile-induced overexpression of genes with oncogenic function in treated human hypopharyngeal primary cells (HHPC) NF-κB inhibition-induced transcriptional levels (with and without BAY 11-7082) of the analyzed NF-κB related genes with oncogenic function, are depicted for bile-treated human hypopharyngeal primary cells (HHPC). The data are derived by real time qPCR analysis. (**A**) Graphs, created by Graph Pad Prism 6 software, reveal transcriptional levels (normalized to *h*GAPDH) for the analyzed genes between different experimental and control groups of treated HHPC (ONE-WAY ANOVA, Freidman test; ^*^p < 0.05; ^**^p < 0.005). (**B**) Graphs represent transcriptional levels of each analyzed gene, bcl-2, EGFR, ΔNp63, c-REL, RELA(p65), TNF-α, STAT3, WNT5α, IL-6 and IL-1β (relative to hGAPDH reference gene), in HHPC treated with and without BAY 11-7082 (^*^p < 0.05; ^**^p < 0.005, ^***^p < 0.0005, ^****^p < 0.00005, by *t*-test; multiple comparisons by Holm-Sidak; GraphPad Prism 6.0). (Data of three independent experiments).

**Figure 6:**
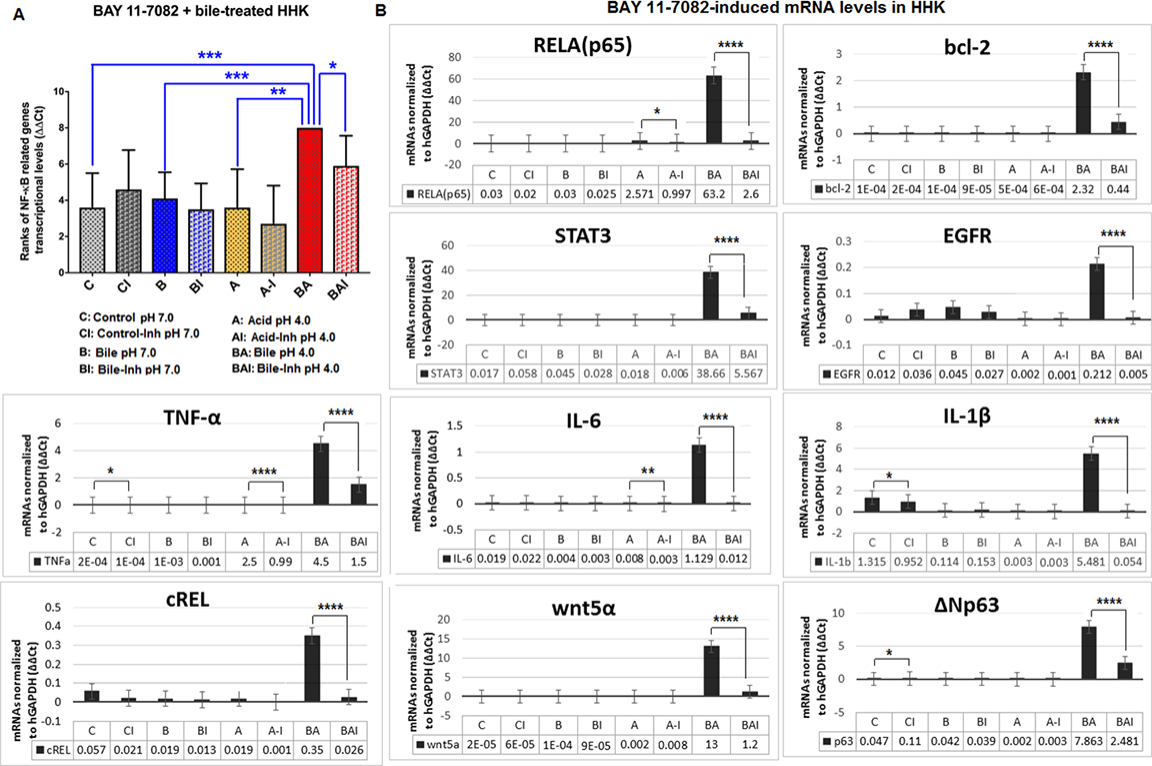
Inhibition of NF-*κ*B prevents the acidic bile-induced overexpression of genes with oncogenic function in treated human hypopharyngeal keratinocytes (HHK) NF-κB inhibition-induced transcriptional levels (with and without BAY 11-7082) of the analyzed NF-κB related genes with oncogenic function, are depicted for bile-treated human hypopharyngeal keratinocytes (HHK). The data are derived by real time qPCR analysis. (**A**) Graphs, created by Graph Pad Prism 6 software, reveal transcriptional levels (normalized to *h*GAPDH) for the analyzed genes between different experimental and control groups of treated HHK (ONE-WAY ANOVA, Freidman test; ^*^p < 0.05; ^**^p < 0.005). (**B**) Graphs represent transcriptional levels of each analyzed gene, bcl-2, EGFR, ΔNp63, c-REL, RELA(p65), TNF-α, STAT3, WNT5α, IL-6 and IL-1β (relative to hGAPDH reference gene), in HHK treated with and without BAY 11-7082 (^*^p < 0.05; ^**^p < 0.005, ^***^p < 0.0005, ^****^p < 0.00005, by *t*-test; multiple comparisons by Holm-Sidak; GraphPad Prism 6.0). (Data of three independent experiments).

We performed real time qPCR analysis on whole transcriptomes of acidic bile-treated normal human hypopharyngeal cell cultures exposed to BAY 11-7082. The acidic bile treated groups, without NF-κB inhibitor, demonstrated the highest transcriptional levels of the analyzed NF-κB related genes with oncogenic function (Figures 5 and 6). Specifically, we observed that acidic bile groups without BAY 11-7082 produced a statistically significant difference compared to acid alone (pH 4.0), bile at pH 7.0 and neutral control (pH 7.0), in both HHPC (*p* = 0.0106, *p* = 0.0137 and *p* < 0.0001, respectively) and HHK (*p* < 0.0001, *p* = 0.0007 and *p* = 0.0005, respectively), in line with our prior studies [21, 22] (by Friedman test; Dunns’ multiple comparisons). BAY 11-7082 prevented bile-induced transcriptional activation of the analyzed genes at pH 4.0, both in treated HHPC (Figure 5) and HHK (Figure 6). Specifically, we observed significantly lower transcriptional levels of the analyzed genes in HHPC and HHK treated with bile at pH 4.0 plus BAY 11-7082, compared to those treated by acidic bile without inhibitor (*p* = 0.0047 and *p* = 0.0168, respectively) (by Friedman test) (Figures 5A and 6A).

We found that BAY 11-7082 inhibited the acidic bile-induced overexpression of all the analyzed genes. However, RELA, STAT3, EGFR, bcl-2 and IL-1β were most affected genes by BAY 11-7082, in both acidic bile-treated HHPC and HHK (Figures 5B and 6B). This was demonstrated by the strong differential expression of the analyzed genes with and without NF-κB inhibitor (*p* values < 0.0001; by *t*-test) (Figures 5 and 6; see Supplementary Tables 2 and 3). We also noticed that BAY 11-7082 reduced the c-REL mRNA levels both in neutral bile (pH 7.0) and acidic bile (pH 4.0) conditions, in HHPC (Figure 5B).

**Figure 7:**
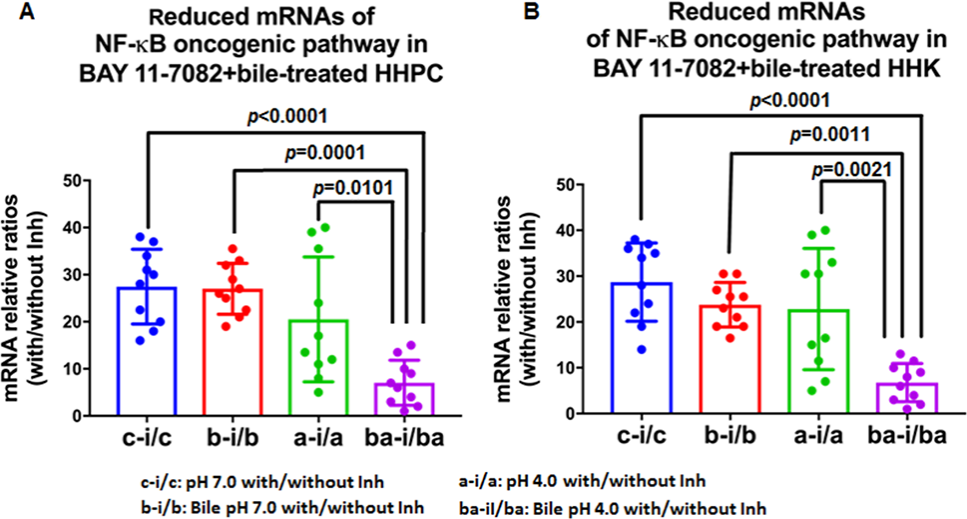
Acidic bile-treated groups in the presence of BAY 11-7082 produced the most significant mRNA reduction of NF-κB related genes with oncogenic function in treated normal human hypopharyngeal cells Graphs created by Graph Pad Prism 6.0 software reveal ranks of transcriptional levels (with/without NF-κB inhibitor) of NF-κB related genes with oncogenic function between different experimental and control groups, in treated (**A**) human hypopharyngeal primary cells (HHPC) and (**B**) human hypopharyngeal keratinocytes (HHK), by real time qPCR. (ONE-WAY ANOVA, Kruskal-Wallis).

We performed multiple comparisons between the estimated ratios of relative transcriptional levels, with/without NF-κB inhibitor, in acidic bile-treated HHPC and HHK, and control groups (Figure 7A and 7B). We observed that the acidic bile-treated group was the most affected by NF-κB inhibitor. We showed that HHPC and HHK treated with bile at pH 4.0 exhibited significantly lower mRNA ratios (with/without BAY 11-7082), compared to neutral-control (*p* < 0.00001 and *p* < 0.0001, respectively), bile at pH 7.0 (*p* = 0.0001 and *p* = 0.0011, respectively) and acid alone (*p* = 0.01 and *p* = 0.0021, respectively) (by Kruskal-Wallis).

Both HHK and HHPC exposed to vehicle (DMSO) resulted in similar mRNA levels to neutral-control (see Supplementary Figure 2 and Supplementary Table 4 online). We observed that only ΔNp63 and IL-1β demonstrated significantly lower mRNA ratios in DMSO-treated HHK compared to control (Supplementary Figure 2B) (*t*-test, *p* values < 0.05).

**Figure 8:**
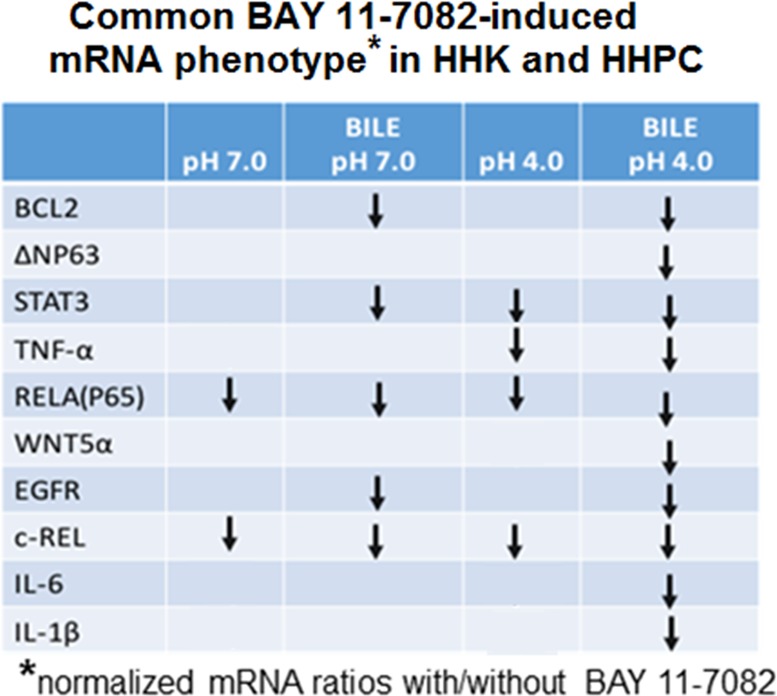
BAY 11-7082-induced common mRNA phenotype in treated normal human hypopharyngeal cells Table describes BAY 11-7082-induced common mRNA phenotypes (relative reduced mRNA levels of genes in cells exposed to BAY 11-7082 compared to those treated without BAY 11-7082) in bile at pH 4.0, bile at pH 7.0, acid at pH 4.0 and control at pH 7.0 treated normal human hypopharyngeal cells (HHPC and HHK). We demonstrate that the acidic bile group produces a reduced mRNA phenotype for all analyzed genes in the presence of BAY 11-7082. In contrast, acid alone (pH 4.0), bile at pH 7.0 and control at pH 7.0 treated groups demonstrate a reduced mRNA phenotype only in a part of the analyzed genes.

Figure 8 shows that NF-κB inhibition down-regulates the acidic bile-induced mRNA phenotype, including all analyzed genes. A less intense effect of NF-κB inhibition is observed in mRNA phenotypes of normal human hypopharyngeal cells treated by bile at pH 7.0 and controls (pH 7.0 and pH 4.0), suggesting that only a part of the analyzed genes is affected by BAY 11-7082.

### Correlations between BAY 11-7082 induced transcriptional levels of NF-κB and related genes in HHK and HHPC

We performed a *Pearson* analysis to determine correlations between the transcriptional levels of NF-κB transcriptional factor RELA(p65) and NF-κB related genes in normal human hypopharyngeal cells exposed to NF-κB inhibitor (see Supplementary Figure 3 online). We found, in both treated HHK and HHPC, a significant linear correlation between BAY 11-7082 induced mRNAs of (i) RELA(p65) and TNF-α (*r* = 0.926192 and *r* = 0.921747, respectively; *p* < 0.05), as well as, (ii) RELA(p65) and ΔNp63 genes (*r* = 0.915 and *r* = 0.933285, respectively; *p* < 0.05).

We also observed, in HHK, a strong positive correlation between BAY 11-7082 induced mRNAs of (i) RELA(p65) and IL-1β (*r* = 0.924554; *p* < 0.05), (ii) RELA(p65) and IL-6 (*r* = 0.920726; *p* < 0.05), (iii) RELA(p65) and STAT3 (*r* = 923810772, p < 0.5), and (iv) RELA(p65) and bcl-2 (*r* = 0.92646569, *p* < 0.05).

We performed a *Pearson* analysis between BAY 11-7082 induced mRNA levels of the NF-κB related cytokines, IL-1β and IL-6, and each of the analyzed NF-κB related genes in treated cells (see Supplementary Figure 4 online). In HHK, we observed a strong linear correlation between (i) IL-1β and TNF-α (*r* = 0.992982; *p* < 0.05), (ii) IL-6 and TNF-α (*r* = 0.998645; *p* < 0.05), and (iii) IL-1β and ΔNp63 (*r* = 0.999548; *p* < 0.05). Similarly, in HHK, we observed a strong linear correlation between (i) IL-6 and ΔNp63 (*r* = 0.998349; *p* < 0.05), (ii) IL-1β and STAT3 (*r* = 0.992072, *p* < 0.05), and (iii) IL-6 and STAT3 (*r* = 0.998183, *p* < 0.05). Finally, in HHK, we again showed a significant linear correlation between (i) IL-1β and WNT5A (*r* = 0.993047, *p* < 0.05), (ii) IL-6 and WNT5A (*r* = 0.998653, *p* < 0.05), as well as between (iii) IL-1β and IL-6 (*r* = 0.9997677574, *p* < 0.5).

### BAY 11-7082 reduced acidic bile-induced gene expression profiling for NF-κB signaling pathway in normal human hypopharyngeal cells

Our findings from qPCR analysis demonstrated that BAY 11-7082 induced the most significant reduction of activated NF-κB and related genes in the acidic bile-treated groups. Therefore, we performed a PCR array for the NF-κB signaling pathway to explore acidic bile-induced gene expression profiling of NF-κB signaling inhibition by BAY 11-7082. The results of the PCR array demonstrated that BAY 11-7082 reduced the acidic bile-induced transcriptional levels of 72 out of 84 analyzed NF-κB-related genes (∼85%) (> 2-fold change) (Table 1).

**Table 1:** Down-regulation of NF-κB signaling pathway in acidic bile with BAY 11-7082 treated normal human hypopharyngeal cells

Gene	^*^Fold regulation	Gene	Fold regulation	Gene	Fold regulation
AGT	-16.1537	HMOX1	-28.5629	RAF1	-1.9048
AKT1	-18.3971	ICAM1	-16.1537	REL	-30.4768
ATF1	-10.5717	IFNA1	-20.6671	RELA	-13.0886
BCL10	-258.858	IFNG	30.6027	RELB	-6.6377
BCL2A1	-31.7151	IKBKB	-192.093	RHOA	11.7111
BCL2L1	-23.4387	IKBKE	-16.1537	RIPK1	-16.1537
BCL3	-33.8756	IKBKG	-16.1537	STAT1	-22.2708
BIRC2	-5.1434	IL10	-16.1537	TBK1	-19.7758
BIRC3	-11.5315	IL1A	-16.1537	TICAM1	-16.5324
CARD11	-28.861	IL1B	-49.4878	TICAM2	-16.1537
CASP1	-807.1	IL1R1	-34.9508	TIMP1	-230.741
CASP8	-807.1	CXCL8	-29.7664	TLR1	-59.8221
CCL2	-16.1537	IRAK1	-16.1537	TLR2	-94.5984
CCL5	-1.5542	IRAK2	-15.0469	TLR3	-1.5561
CD27	-22.2086	IRF1	-250.692	TLR4	-15.56
CD40	-1.1076	JUN	-250.692	TLR6	-4802.17
CFLAR	-19.0453	LTA	-16.1537	TLR9	-1.3347
CHUK	-293.394	LTBR	4.039	TNF	-1366.74
CSF1	-472.978	MALT1	-46.6225	TNFAIP3	-54.5169
CSF2	-11.1574	MAP3K1	-227.97	TNFRSF10A	-109.034
CSF3	-22.3147	MYD88	-8.1661	TNFRSF10B	-51.1432
EGFR	-7.6029	NFKB1	-2723.64	TNFRSF1A	-12.1615
EGR1	-7.6029	NFKB2	-131.744	TNFSF10	-10.3132
ELK1	-16.1537	NFKBIA	3.3532	TNFSF14	-16.1537
F2R	-12.5571	NFKBIB	26.0694	TRADD	-34.034
FADD	-25.1141	NFKBIE	-22.2708	TRAF2	-42.32
FASLG	4.8938	NOD1	20.0837	TRAF3	-174.151
FOS	-1073.93	PSIP1	-50.9866	TRAF6	-31.2479

^*^Up-Down Regulation in acidic bile with BAY 11-7082 (group 1), comparing to acidic bile without BAY 11-7082 treated normal human hypopharyngeal cells (control group).

**Figure 9:**
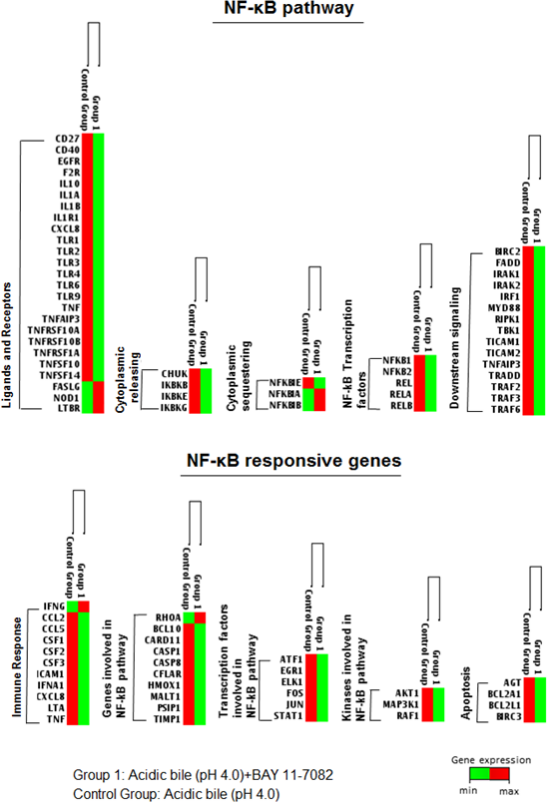
BAY 11-7082 reduced the acidic bile-induced gene expression profiling of NF-κB signaling pathway in treated normal human hypopharyngeal cells Heat maps were obtained by RT^2^-Profiler PCR array analysis for NF-κB signaling. The genes were clustered based on their biological role. The heat maps demonstrated the effect of BAY 11-7082 in gene expression of NF-κB pathway and NF-κB responsive genes (red color for maximum expression and green for minimum). Group 1: Acidic bile-treated cells with BAY 11-7082; Control Group: Acidic bile-treated cells without BAY 11-7082. (Gene expression has been normalized to two housekeeping genes; hGAPDH, human glyceraldehyde-3-phosphate dehydrogenase and RPLP0, ribosomal protein lateral stalk subunit P0).

The effect of BAY 11-7082 on the NF-κB pathway and its genes is provided in Figure 9. NF-κB inhibitor reduced the mRNA levels of the NF-κB transcription factors, RELA(p65) (13-fold), RELB (> 6-fold), and NFkB1 (> 1,000-fold), NFkB2 (> 130-fold), as well as members of TNF-receptors, such as CD27(TNFRS7) (> 22-fold), TNFRSF10A (> 100-fold), TNFRSF10B (> 50-fold), TNFRSF1A (> 12-fold), TNFSF10 (> 10-fold) and TNFSF14 (> 14-flod), TLR1 (> 50-fold), TLR-2 (> 90-fold), TLR4 (> 15-fold) TLR6 (> 1,000-fold), as well as IL10 and IL1A (> 16-fold) and others. BAY 11-7082 also reduced NF-κB downstream signaling, preventing the expression of positive regulators of the NF-κB pathway, such as BIRC2 (> 5-fold), IRAK1 and IRAK 2 (> 15-fold), IRF1 (> 250-fold), MYD88 (> 8-fold), TBK1 (> 19-fold), TRAF2, TRAF3 and TRAF6 (> 30-fold). The effect of BAY 11-7082 also reduced the expression of Inhibitor-kappaB kinases, CHUK (IKKa) (> 290-fold), and IKBKB, IKBE and IKBKG (> 16-fold), inhibiting the cytoplasmic release of NF-κB. On the other hand, BAY 11-7082 induced the expression of NFKBIA (> 3-fold) and NFKBIB (> 26-fold), keeping NF-κB protein complexes sequestered in an inactive state in the cytoplasm.

NF-κB inhibition resulted in reduction of NF-κB responsive genes in acidic bile treated cells. Specifically, we observed a reduction in the expression of genes, such as kinase MAP3K1 (> 220-fold), and transcriptional factors, such as ATF1 (> 10-fold), EGR1 (> 7-fold), ELK1 (> 16-fold), FOS (> 1,000-fold) and STAT1 (> 20-fold). Finally, we found a high level of reduction in BCL10 (> 250-fold) and CARD11 (> 28-fold), activators of NF-κB through BCL10.

## DISCUSSION

Although there is a heterogeneity of molecular profiles in head and neck cancer, Cheng and Van Waes have strongly suggested the central role of Nuclear Factor kappaB in a subset of Head and Neck Squamous Cell Carcinoma (HNCC) [31, 32]. NF-κB is a fundamental mediator between inflammation and cancer and its possible role as a mechanistic link between chronic inflammatory events and activation of oncogenic pathways is well recognized [33, 34]. Additionally, inhibition of NF-κB has been recently shown to be inversely related to head and neck tumor progression and invasion [35] promising its use in cancer prevention and therapy [27].

Despite the known risks of tobacco and alcohol consumption in head and neck carcinogenesis, there is an increasing clinical correlation between laryngopharyngeal reflux or LPR and laryngopharyngeal neoplasia [4–6, 36, 37]. Prior findings from our *in vitro* and *in vivo* models demonstrated that acidic bile can induce NF-κB activation and accelerate the transcriptional levels of genes related to oncogenic function [21, 22]. We previously have shown that bile and acid in combination contributed significantly to NF-κB activation and bcl-2 overexpression *in vitro*, in treated normal hypopharyngeal cells, and *in vivo*, in murine hypopharyngeal mucosa, relative to acid alone or other factors such as topical glucose or pepsin [21–23]. We showed *in vivo* that this combination induced premalignant hypopharyngeal lesions, exhibiting increased cell proliferation rates and transcriptional activation of genes with anti-apoptotic or oncogenic function, such as EGFR, STAT3, TNF-α, wnt5A. The negative or reduced effect of acid alone and/or bile at neutral pH, compared to acidic bile salts showed that the latter may be especially injurious. Here, we explored the inhibition of NF-κB in preventing the acidic bile-induced mRNA phenotype, including previously analyzed NF-κB transcriptional factors, such as RELA(p65) and c-REL, anti-apoptotic or oncogenic factors, bcl-2, EGFR, STAT3, and ΔNp63, cell signaling factor TNF-α, NF-κB related cytokines IL-1β and IL-6, and epithelial mesenchymal transition (EMT) mediator WNT5A. We further explored the effect of BAY 11-7082 on the inhibition of the acidic bile-induced transcriptional activation of NF-κB signaling pathway.

Our novel findings demonstrate that NF-κB inhibition, by BAY 11-7082, effectively prevents the acidic bile-induced overexpression of the analyzed genes, in treated normal human hypopharyngeal cells. BAY 11-7082 effectively reduced the acidic bile-induced expression of 72 genes related to NF-κB signaling pathway. These findings strongly support our hypothesis that the acidic bile-induced mRNA phenotype is mainly produced by NF-κB-related signaling pathways and that NF-κB may be a critical link between acidic bile and preneoplastic events in our model.

TNF-α is a major inflammatory cytokine with a key role in cancer [15, 38], and with NF-κB central to its activation in HNSCC [17]. Previous studies demonstrate that NF-κB is capable of directly or indirectly activating oncogenic pathways via cytokines, such as IL-6 and IL-1β, in HNSCC [13, 18]. Our data demonstrate that NF-κB inhibition can block the acidic bile-induced overexpression of TNF-α, NF-κB transcriptional factor RELA(p65), and cancer related cytokines, IL-1β and IL-6. Our analysis reveals a strong association between BAY 11-7082-induced transcriptional levels of RELA(p65) and TNF-α or the analyzed cytokines. We believe these observations suggest that NF-κB inhibition is capable of blocking acidic bile-induced downstream pathways that link inflammation to cancer.

The anti-apoptotic role of NF-κB [35, 39], as well as interactions of NF-κB with oncogenic factors, such as EGFR and STAT3 have been previously cited in HNSCC [11, 39, 40]. STAT3 is an important factor with oncogenic function in head and neck malignancies [13]. EGFR is also broadly expressed in head and neck cancer and has been linked to patient survival [11, 13]. Our data demonstrate that BAY 11-7082 effectively prevents the acidic bile-induced overexpression of anti-apoptotic bcl-2, and oncogenic EGFR and STAT3, in normal human hypopharyngeal cells. These observations strongly support the role of BAY 11-7082 in effectively diminishing the proposed oncogenic effect of acidic bile.

c-REL is an important member of NF-κB family and acts as an oncoprotein in HNSCC through interactions with ΔNp63, via the TNF-α pathway [41]. Yang et al. reveal that ΔNp63 is a master transcription factor that in coordination with NF-κB/RELs, promotes inflammation and the malignant phenotype of HNSCC [42]. Our current data show that NF-κB Inhibition significantly prevents c-REL expression, particularly in human hyopharyngeal cells exposed to bile at pH 4.0. We also show that inhibition of NF-κB reduces ΔNp63 transcriptional levels, while BAY 11-7082-induced RELA(p65) and ΔNp63 mRNA levels demonstrate a strong linear correlation. These findings further demonstrate that NF-κB inhibition can affect acidic bile-induced inflammatory and cell proliferation signaling pathways.

WNT5A, linked to the epithelial mesenchymal transition (EMT) process and cancer progression [43], has been shown to be upregulated by NF-κB signaling [44]. Furthermore, our prior *in vivo* model showed that acidic bile is capable of altering both the expression of WNT5A and the cell-cell adhesion molecules, β-catenin and E-Cadherin [22]. This interaction was observed in treated murine laryngopharyngeal mucosa that exhibited premalignant changes with strong NF-κB activation [22]. Here our data show that NF-κB inhibition suppresses the acidic bile-induced overexpression of WNT5, in treated cells, supporting the possibility that inhibition of NF-κB may offer protection from changes associated with cell-cell interactions.

Finally, our data show a strong positive correlation between BAY 11-7082-induced transcriptional levels of RELA(p65) and oncogenic STAT3 or WNT5A, confirming previously suggested interactions among these factors, in acidic bile-treated normal human hypopharyngeal cells [21].

## MATERIALS AND METHODS

### Normal human hypopharyngeal keratinocyte cultures

We used human hypopharyngeal primary cells (HHPC) from Celprogen Inc. CA, USA. The HHPC were plated in non-coated flasks and were grown in Human Hypopharyngeal Normal Cell Culture Media with Serum (Celprogen Inc. CA, USA), at 37°C in humidified air and 5% CO2. The HHPC were sub-cultured and media were gradually replaced by Serum Free Media (Celprogen Inc. CA, USA), and passed after reaching ∼90% confluence, using 0.05% trypsin-EDTA (Gibco^®^, NY, USA).

We also established a telomerase-immortalized human hypopharyngeal keratinocytes (HHK), by expression of hTERT, extending its life span without altering the characteristic phenotypic properties of the cells, as previously described [20]. HHK, were grown in keratinocyte serum free basal medium (KGM-2 SF, Gibco^®^, NY, USA) supplemented by L-Glutamine, BPE, hEGF and gentamicin (Gibco^®^, NY, USA), at 37°C in humidified air and 5% CO2.

### Treatment conditions

*Bile-treatment:* HHPC (2nd passage) and the HHK (4th passage) underwent a repetitive exposure to experimental and control media for 10–15 min, 3 times per day, for 5 days, as previously described [21]. Experiments were performed in triplicate for each cell line. In each assay HHPC and HHK were treated after 2nd and 4th passage, respectively.

Experimental groups included a repetitive exposure to bile at pH 4.0, the cut off of reflux disease [45], and at pH 7.0, as follows: (a) bile at pH 4.0, containing 400 μM of conjugated bile salts mixture (GCA+TCA+GCDCA+TCDCA+GDCA+TDCA, Sigma, St. Louis, MO and Calbiochem, San Diego, CA; USA) at molar concentration (20:3:15:3:6:1) as previously described [21], in full growth medium (Dulbecco modified Eagle’s medium/F12 10% FBS, 1% pen/strep, Gibco^®^, NY, USA), brought to a pH of 4.0 with 1M HCl (using a pH meter) and (b) bile at pH 7.0, containing the same bile salts mixture in DMEM/F12 10% FBS, at pH 7.0.

Control groups included a repetitive exposure to acid alone (pH 4.0) considered a positive control and neutral fluid (pH 7.0) considered a reference control, as follows: (a) Acid control, full growth DMEM/F12 10% FBS, brought to pH 4.0 with 1M HCl, and (b) neutral control, full growth DMEM/F12 10% FBS, pH 7.0. The media were removed and replaced with serum free media until the next exposure cycle [Human Hypopharyngeal Normal Cell Culture Media Serum Free, for HHPC cells; (Celprogen Inc. CA, USA), and KGM-2 SF, for HHK cells (Gibco^®^, NY, USA)].

*BAY-11-7082 treatment:* HHPC and HHK underwent an additional procedure of combined repetitive exposure to bile with BAY-11-7082, a pharmacologic inhibitor of NF-κB (Calbiochem © 2016 EMD Millipore Corporation; Germany), for 10-15 min, 3 times per day, for 5 days.

Experimental groups included an identical procedure of repetitive exposures of HHPC and HHK to bile at pH 4.0 or pH 7.0, as described above, in combination with BAY 11-7082, as follows: (a) bile at pH 4.0, containing 400 μM of conjugated bile mixture plus 10 μM of BAY 11-7082, in full growth medium, at pH 4.0 and (b) bile at pH 7.0 plus BAY 11-7082, containing the same bile salts and NF-κB inhibitor mixture, in full growth medium, at pH 7.0. We selected a concentration of 10 μM of BAY 11-7082 in accordance with previous reports [46].

Control groups included repetitive exposure to acid alone (pH 4.0) and neutral fluid (pH 7.0) in combination with BAY 11-7082, as follows: (a) Acid control, full growth medium, as described above, plus 10 μM of BAY 11-7082, at pH 4.0 that was used as positive control and (b) neutral control, full growth medium, as described above, plus 10 μM of BAY 11-7082, at pH 7.0. We also included additional control groups of untreated cells, used as negative control and groups of cells repetitively exposed to DMSO, at concentrations similar to those used for BAY 11-7082 solubilisation used as reference control for the NF-κB inhibitor vehicle. Cells of experimental and control groups that were treated without inhibitor did not include a vehicle control. The media were removed and replaced with serum free media until the next exposure cycle, as described above.

Procedures on both cell lines were performed in parallel and at the end of treatment media were removed and cells or cell extracts were analysed.

### Immunofluorescence assay

We performed an immunofluorescence assay to explore the effect of NF-κB inhibitor on the acidic bile-induced nuclear translocation of NF-κB transcription factor p65, phosphorylated at Ser536, linked to NF-κB activation mediated by IKKb and/or IKKa [46]. Cytokeratin 14 (CK14) was used to reveal the cytoplasmic cellular compartment of the treated cells. HHPC were grown on slides (multiwall chamber slides; Lab-Tek^®^) and underwent repeated exposure with experimental and control fluids with or without NF-κB inhibitor (10 μM of BAY 11-7082). At the end of treatment, cells were fixed immediately after the final treatment in 4% paraformaldehyde (Sigma-Aldrich) for 7 minutes and incubated with 1:65 of primary anti-NF-κB (rabbit polyclonal anti-phospho-p65 Ser536, AbD Serotec, BIO-RAD, CA, USA), overnight at 4°C, after permeabilization of cell membranes using 0.2% Triton X100 (AmericanBio, Natick, MA, USA) in PBS for 2–3 minutes and blocking with 2% bovine serum albumin (BSA) in PBS (Sigma-Aldrich, USA) for 1 hour. The next day, cells first were washed in 1% Tween20 (AmericanBio, Natick, MA, USA) in PBS for 1–2 min, then with 0.1% BSA in PBS twice for 5 min and subsequently were incubated with 1:500 dilutions of secondary anti-rabbit DyLight^®^488 (green; Vector Labs, USA), for 1 hour, at room temperature. Cells were washed and incubated with 1:50 primary anti-cytokeratin 14 (mouse monoclonal Ab, LL002, abcam^®^, MA, USA) overnight at 4°C. The next day cells were washed and incubated with 1:500 secondary anti-mouse DyLight^®^650 (red; Vector Labs, USA), for 1 hour, at room temperature, washed and mounted using Prolong Gold Mountant with diamidino-phenylindole (ProLong^®^ Diamond Antifade Mountant with DAPI; Life Technologies, Thermo Scientific, MA, USA) for nuclear staining and mounting of cells (blue). The slides were examined using a Zeiss Confocal microscope and images were captured and analysed using Zen imaging software from Carl Zeiss, microscopy (Germany). Total p-p65 (S536) expression levels in treated HHPC with/without NF-κB inhibitor were identified by fluorescence intensity (mean±SD bin count) of two independent images (≥10 cells) (Zen imaging software).

### Western blotting

We performed western blot analysis, as described previously and included in the Supplementary information online, to determine the protein expression levels of NF-κB (p65), phospho-inhibitor kappaB-α (p-IκB-α) and bcl-2, in treated human hypopharyngeal primary cells (HHPC) and human hypopharyngeal keratinocytes (HHK) with and without NF-κB inhibitor, BAY 11-7082. Specifically, we isolated cytoplasmic and nuclear fractions from treated cells at the end of three independent experiments, using NE-PER nuclear and cytoplasmic extraction, including protease inhibitors (Thermo Scientific, Pierce, NY). We measured the total cytoplasmic and nuclear protein concentrations of cell extracts using the BCA-200 Protein Assay kit (Thermo Scientific). We used anti-NF-κB (rabbit polyclonal anti-phospho-p65 Ser536, AbD Serotec, BIO-RAD, CA, USA)), phospho-IκB-α Ser32/36 (5A5; Cell Signaling, EMD Millipore, Billerica, MA), and bcl-2 (C-2; Santa Cruz Biotechnology) and β-actin (C4; Santa Cruz Biotechnology) for cytoplasmic extracts and Histone 1 (AE-4; Santa Cruz Biotechnology) for nuclear extracts normalization. Protein levels were quantified by Gel imaging system (*BIO-RAD*) in each nuclear and cytoplasmic cellular compartment, and expression levels were estimated by Image Lab 5.2 analysis software (*BIO-RAD*), as described in Supplementary information online.

### Luciferase assay

We performed a luciferase assay in order to monitor the activity of the NF-*κ*B in HHPC and HHK exposed to bile with or without the pharmacologic inhibitor of NF-κB, BAY 11-7082. We used Dual-Glo^®^ Luciferase Assay system (Promega Corporation, Madison, WI, USA), Lipofectamine^®^ 2000 (Invitrogen™), and a plasmid containing firefly luciferase gene under the control of NF-κB responsive element (NF-κB reporter, 3kB-ConA-luc) or a plasmid containing firefly luciferase gene without any additional response elements (ConA-luc, reference control plasmid) [23], in accordance with the manufacturer’s procedure. The treatment was performed 24 hours after transfection. We performed triplicate assays for each treatment condition (bile with or without NF-κB Inhibitor and corresponding controls, at pH 4.0 and pH 7.0). The cells were treated once for 30 min and followed after 5–6 hours by an additional 10 min treatment. This was performed 2 times/day, constituting a regimen of repetitive exposures. At the end of the final treatment, luminescence was measured using a luminometer (Infinite^®^ M1000 PRO, TECAN) and i-control™ software. We expressed NF-κB activity as ratios of mean values (values for NF-κB reporter, 3kB-ConA-luc, against the mean value for reference control, conA-luc) calculated in treated HHPC and HHK for each condition. Finally, we expressed the alterations of NF-κB activity induced by BAY 11-7082 as ratios of relative NF-κB activity (with/without NF-κB inhibitor).

### Quantitative real time PCR

We isolated total RNA (RNeasy mini kit; Qiagen Inc., CA, USA) from HHK and HHPC exposed to GDF with or without BAY 11-7082, and corresponding controls, to evaluate the transcriptional levels of RELA (p65), c-REL, bcl-2, TNF-α, ΔNp63, EGFR, STAT3, WNT5A, IL-1β and IL-6, using quantitative real time polymerase chain reaction (qPCR) analysis, as previously described [21]. Briefly, we determined RNA quality and concentration by absorption ratios at 260/280 nm (> 2.0) and 260 nm, respectively (NanoDrop™ 1000 spectrophotometer; Thermo Fisher Scientific, Waltham, MA). We performed reverse transcription (iScript cDNA synthesis kit; Bio-Rad) and real time qPCR analysis (Bio-Rad real time thermal cycler CFX96TM; Bio-Rad) using specific primers for target genes and reference housekeeping gene, human glyceraldehyde 3-phosphate dehydrogenase (*h*GAPDH) (Supplementary Table 1; see Supplementary information online), (QuantiTect Primers Assays; Qiagen), and iQ™ SYBR Green Supermix (Bio-Rad). We performed assays in 96-well plates, in triplicate for each sample, and data were analyzed by CFX96™ software. Relative mRNA expression levels were estimated for each target gene relative to reference gene (ΔΔ*C*t). (Data were obtained from three independent experiments)

### Statistical analysis

Statistical analysis was performed using GraphPad Prism 6 software and ONE-WAY ANOVA (Friedman or Kruskal-Wallis and Dunn’s multiple analysis test; *p*-values < 0.05) as well as *t*-test analysis (multiple comparisons by Holm-Sidak) to reveal any evidence of statistically significant reductions in protein or mRNA expression levels in different experimental and control groups treated by BAY 11-7082. We performed *Pearson* correlation to estimate the correlation coefficient between expression levels of different groups (*p*-values < 0.05). Specifically, we used *Pearson* analysis to identify correlations between the BAY 11-7082-induced transcriptional levels of the analyzed NF-κB transcriptional factor, RELA (p65) and NF-κB related genes, in the four different groups of treated HHK and HHPC.

### PCR array for NF-κB signaling pathway

We performed a PCR microarray analysis of the NF-κB signaling pathway in acidic bile treated groups with and without BAY 11-7082 to identify the effect of NF-κB inhibitor on the acidic bile-induced gene expression profiling of the NF-κB signaling pathway. Specifically, we used a transcriptome of human hypopharyngeal keratinocytes and a PCR array kit for human NF-κB signaling pathway (RT^2^-Profiler PCR array, PAHS-025z; SABiosciences, Qiagen), following the manufacturer’s instructions. The data were analyzed online by RT^2^-Profiler PCR Array Data Analysis version 3.5 software and differential expression of more than 2-fold-change of gene expression (up and down regulation) was estimated between the acidic bile-treated group (control) and acidic bile-treated with inhibitor group (Group 1).

### Data availability

The datasets generated during and/or analyzed during the current study are available from the corresponding author on reasonable request.

## CONCLUSIONS

We describe a successful *in vitro* model of NF-κB inhibition, using BAY 11-7082, in acidic bile-treated normal human hypopharyngeal cells. We have demonstrated that the presence of BAY 11-7082 significantly reduces the overexpression of NF-κB transcriptional factors and NF-κB related genes with oncogenic function, such as EGFR, TNF-α and STAT3, as well as IL-1β and IL-6, previously linked to acidic bile in early premalignant hypopharyngeal lesions. Additionally, NF-κB inhibition diminishes the transcriptional activation of anti-apoptotic bcl-2 and cell proliferation marker ΔNp63 and WNT5α linked to epithelial mesenchymal transition pathways.

In summary, our novel findings firstly demonstrate that NF-κB inhibition in our model effectively prevents the acidic bile-induced and cancer-related mRNA phenotype and reduces effects of the acidic bile-induced NF-κB signaling pathway, further demonstrating that NF-κB is a critical link between acidic bile and early preneoplastic events. *In vivo* investigation of NF-κB inhibition in acidic bile-treated hypopharyngeal mucosa, supported by future global molecular sequencing analysis, may provide insights into the effective prevention of the induced premalignant phenotype and deregulated oncogenic signaling pathways, previously linked to HNSCC [36]. In future studies, we will also explore non-pharmaceutical NF-κB inhibitors, such as dietary curcumin and others with considered potential clinical application. These observations may further contribute to prevention or therapeutic interventions of extra-esophageal reflux-related laryngopharyngeal neoplasia.

## SUPPLEMENTARY MATERIALS FIGURES AND TABLES


