# Supplementary material for: Inhibition of NF-κB prevents the acidic bile-induced oncogenic mRNA phenotype, in human hypopharyngeal cells
Source: Oncotarget. 2017 Dec 12;9(5):5876–91. doi: 10.18632/oncotarget.23143 (PMC5814181; doi:10.18632/oncotarget.23143)
Supplement: Supplementary file 1 [file oncotarget-09-5876-s001.pdf]

Inhibition of NF-κB prevents the acidic bile-induced oncogenic mRNA phenotype, in human hypopharyngeal cells

SUPPLEMENTARY MATERIALS

SUPPLEMENTARY TO METHODS

Western blotting

At the end of treatment 10 to 30 μg of HHK and HHPC cytoplasmic and nuclear protein extracts were heated at 70°C for 10 minutes in sodium dodecyl sulfate polyacrylamide gel electrophoresis Laemmli sample buffer (Bio-Rad, Hercules, CA), and were separated using 4–20% Mini-PROTEAN TGX Tris/Glycine pre-cast gels, at 150V for 1 hour, while Precision Plus Prestained Protein Standards (Dual Color or Kaleidoscope, Bio-RAD) were used providing a 10-band ladder (250–10 kD). Proteins were transferred onto a 0.45 mm nitrocellulose membrane, using Trans Blot Turbo transfer system (Bio-Rad), blocked in 5% BSA, for 1 hour, and were incubated with primary antibodies, of primary anti-phospho-NF-κB (p65 S536; rabbit polyclonal anti-phospho-p65 Ser536, AbD Serotec, BIO-RAD, CA, USA), phospho-IκB-α Ser32/36 (5A5; Cell Signaling, EMD Millipore, Billerica, MA), and bcl-2 (C-2; Santa Cruz Biotechnology), which were diluted in 5% BSA, overnight at 4°C. Membranes were incubated for 1:30 hours with goat anti-rabbit or anti-mouse horseradish peroxidase conjugated secondary antibodies (EMD Millipore) at 1:10000 and chemiluminescence was determined using an enhanced chemiluminescence detection system (Clarity Western ECL Substrate, Bio-Rad). Membranes also were stripped using Restore stripping buffer (Pierce) and were reported with β-actin (C4; Santa Cruz Biotechnology) for cytoplasmic extracts and Histone 1 (AE-4; Santa Cruz Biotechnology) for nuclear extracts normalization. Protein levels were quantified by Gel imaging system (BIO-RAD). in each nuclear or cytoplasmic cellular compartment, and expression levels were estimated by Image Lab 5.2 analysis software (BIO-RAD).

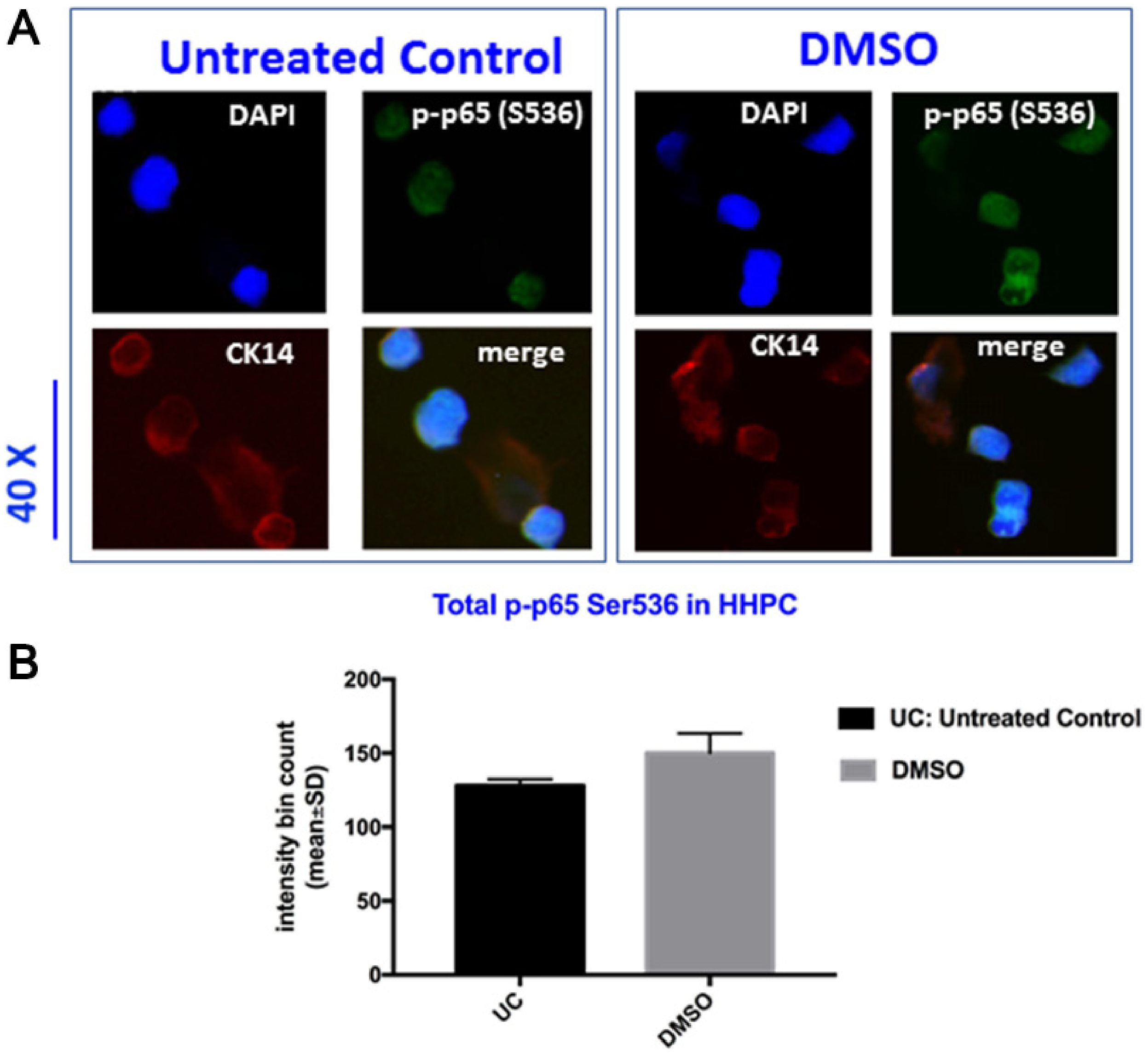

Supplementary Figure 1: Immunofluorescence staining for phospho-NF-κB (p-p65 S536) in DMSO treated and untreated human hypopharyngeal primary cells (HHPC). DMSO does not affect (**A**) nuclear translocation of p-p65 (Ser536), and (**B**) total p-p65 (Ser536) levels, in DMSO treated human hypopharyngeal primary cells (HHPC), similar to untreated HHPC p-p65 nuclear and total levels (green: p-p65 S536; red: CK14 for cytoplasmic staining; blue: DAPI for nuclear staining). (by t-test; mean ± SD; multiple comparisons by Holm-Sidak; GraphPad Prism 6.0).

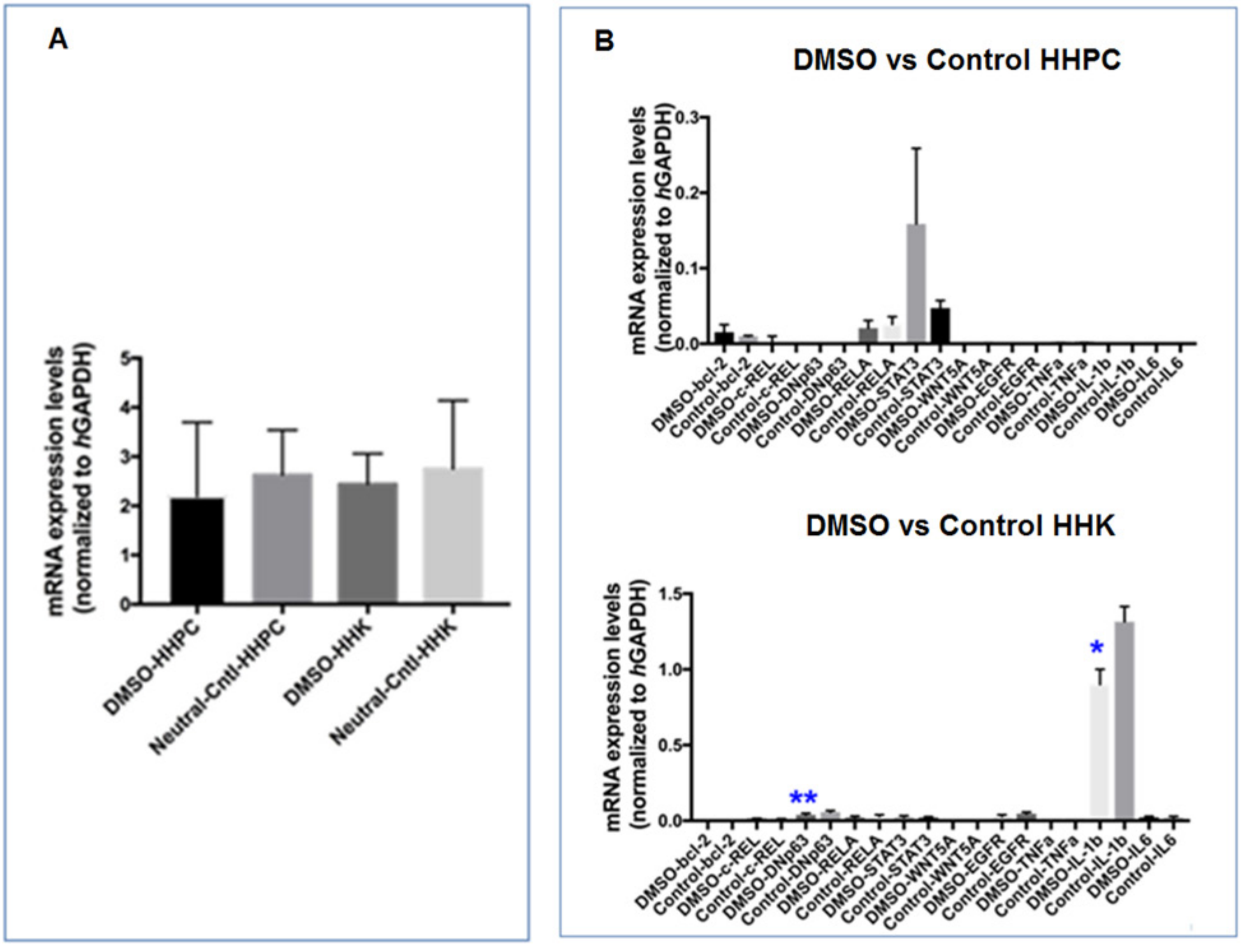

Supplementary Figure 2: qPCR analysis revealed that normal human hypopharyngeal cells exposed to DMSO did not demonstrate any significant changes in the transcriptional levels of analyzed genes compared to corresponding controls. (**A**) Graphs created by Graph PadPrism 6.0 software depict mRNA levels of the analyzed genes treated by DMSO and corresponding Controls (ONE-WAY ANOVA, Freidman test; p value < 0.05; Graph PadPrism 6.0). (**B**) Graphs created by Graph PadPrism 6.0 software depict the transcriptional levels of each analyzed gene in DMSO-treated human hypopharyngeal primary cells (HHPC) and human hypopharyngeal keratinocytes (HHK). The DMSO-treated HHPC and HHK show similar patterns to neutral control mRNA levels for each analyzed gene. ΔNp63 and IL-β are the only genes to demonstrate significantly lower mRNAs in DMSO-treated HHK compared to neutral control (by t-test, GraphPad Prism 6; $^{*}p < 0.05$; $^{**}p < 0.005$).

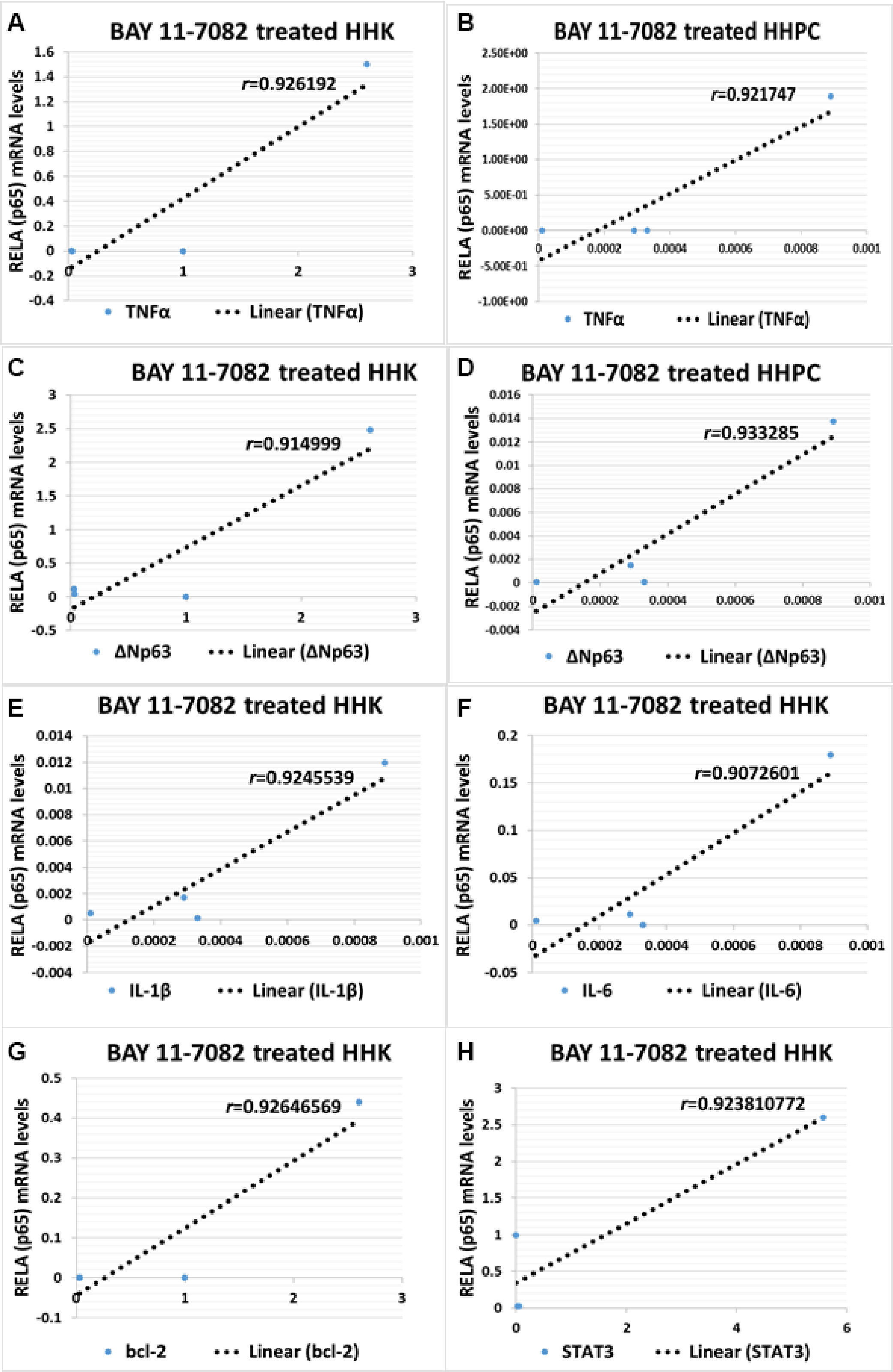

Supplementary Figure 3: Correlation by *Pearson* between BAY 11-7082-induced mRNA levels of RELA(p65) and NF-κB related genes, in treated HHK and HHPC. Diagrams depict a strong linear correlation by Pearson between BAY 11-7082-induced mRNAs of RELA(p65) and TNF-α (**A**, **B**) or ΔNp63 (**C**, **D**), in both HHK and HHPC. A significant positive correlation is also demonstrated between RELA(p65) and cytokines IL-1β (**E**) and IL-6 (**F**), as well as between RELA(p65) and bcl-2 (**G**) or STAT3 (**H**), in treated HHK (p value < 0.05).

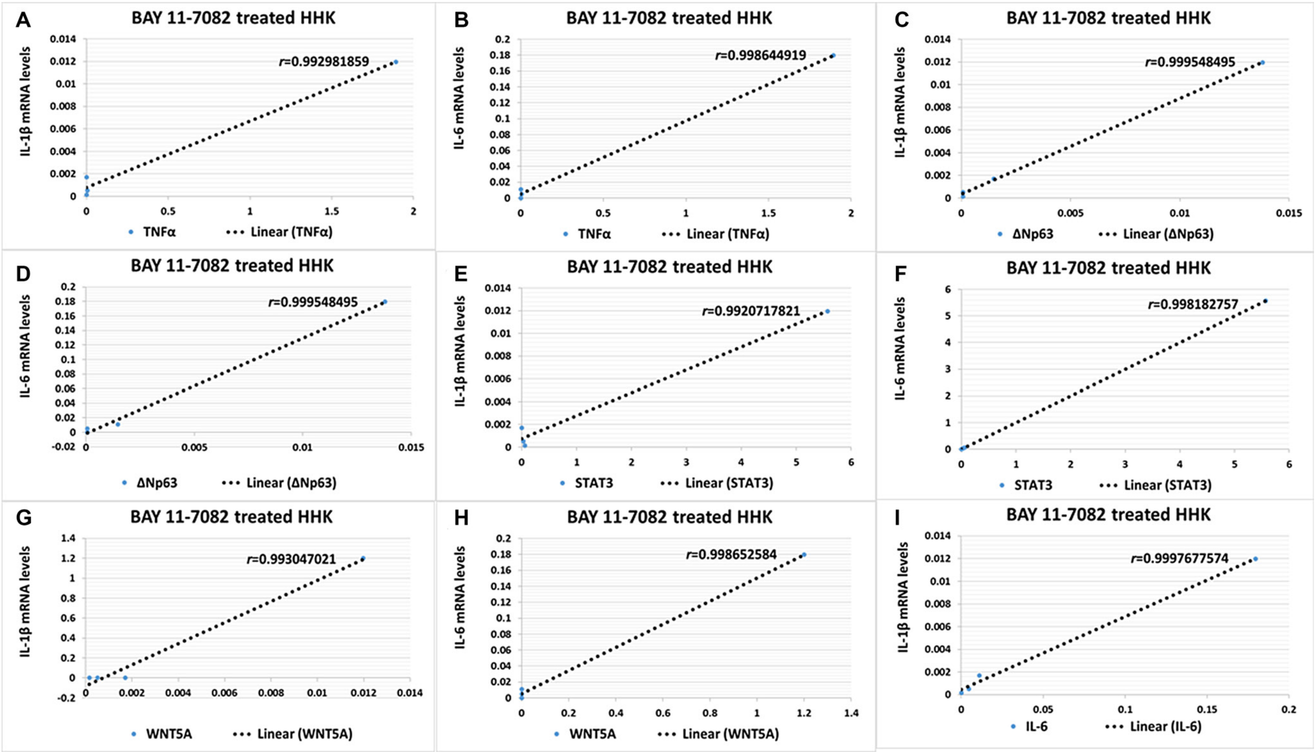

Supplementary Figure 4: Correlation by Pearson between BAY 11-7082-induced mRNA levels of NF-κB related cytokines, IL-1β and IL-6, and each of TNF-α, ΔNp63, STAT3 and WNT5A, as well as between IL-1β and IL-6, in treated HHK. Diagrams depict a strong linear correlation by Pearson between BAY 11-7082-induced mRNAs of cytokines, IL-1β or IL-6, and TNF-α (**A, B**), ΔNp63 (**C, D**), STAT3 (**E, F**), or WNT5A (**G, H**), as well as between IL-1β and IL-6 (I), in treated HHK (p value < 0.05).

Supplementary Table 1: Human genes analyzed by real-time qPCR, in normal human hypopharyngeal cells

Gene	Detected transcripts	Amplicon length (bp)
***h*GAPDH**	NM_001256799, NM_002046	95
**bcl-2**	NM_000633	116
**EGFR**	NM_005228<br>NM_201282-4,	80
**REL**	NM_002908	117
**RELA**	NM_001145138, NM_001243984-5,<br>NM_021975	107
**wnt5A**	NM_001256105, NM_003392	105
**Tp63**	NM_001114980, NM_003722	130
**TNF**	NM_000594	98
**STAT3**	NM_003150,<br>NM_139276	95
**IL-1β**	**NM_000576, XM_006712496**	117
**IL-6**	NM_000600<br>XM_005249745	107

Supplementary Table 2: Down-regulation of NF-κB-related oncogenic pathway in acidic bile with BAY 11-7082 treated human hypopharyngeal primary cells

**Target gene**	**HHPC**			
	**Fold regulation (*mRNA ratios with/without BAY 11-7082)**			
	**Control (pH 7.0)**	**Bile (pH 7.0)**	**Acid (pH 4.0)**	**Bile (pH 4.0)**
**bcl-2**	-1.8	-2.2	2.6	-243
**ΔNp63**	2.3	1.9	11	-398
**STAT3**	10.2	-7	-453	-3739
**TNF-α**	6.3	-7.6	-21	-95
**RELA(p65)**	-7.8	-8.2	-11	-1048
**wnt5A**	-7	-3.2	-115	-19
**EGFR**	-10	-5	22.3	-6918
**c-REL**	-14	-3	-6.2	-6633
**IL-6**	-2.9	2.6	-30	-21
**IL-1β**	1.0	1.1	-22	-49

*Relative mRNA expression ratios (with/without BAY 11-7082) of the analyzed genes were normalized to hGAPDH, by qPCR.

Supplementary Table 3: Down-regulation of NF-κB-related oncogenic pathway in acidic bile with BAY 11-7082 treated human hypopharyngeal keratinocytes

	HHK			
	Fold regulation (*mRNA ratios with/without BAY 11-7082)			
**Target gene**	**Control (pH 7.0)**	**Bile (pH 7.0)**	**Acid (pH 4.0)**	**Bile (pH 4.0)**
**bcl-2**	2.1	-1.3	1.3	-5.3
**ΔNp63**	2.4	-1	1.6	-3.2
**STAT3**	3.4	-1.6	-3.2	-7
**TNF-α**	-1.6	1.1	-2.5	-3
**RELA(p65)**	-1.5	-1.2	-12	-24
**wnt5A**	2.8	1.3	3.5	-11
**EGFR**	2.9	-1.7	-1.7	-42.4
**c-REL**	-2.7	-1.5	-20	-13.2
**IL-6**	1.2	-1.6	3.8	-90.4
**IL-1β**	-1.4	1.3	1.3	-101

*Relative mRNA expression ratios (with/without BAY 11-7082) of the analyzed genes were normalized to hGAPDH, by qPCR.

Supplementary Table 4: Relative mRNA expression of NF-κB-related oncogenic pathway in DMSO-treated normal human hypopharyngeal cells compared to controls

	*mRNA ratios DMSO/Control (pH 7.0)	
**Target gene**	**HHK**	**HHPC**
**bcl-2**	2.5	1.5
**ΔNp63**	-1.3	1
**STAT3**	1.3	2.9
**TNF-α**	1	1
**RELA(p65)**	-1.2	-1.2
**wnt5A**	1.5	-1.3
**EGFR**	1.2	-1.2
**c-REL**	1.1	1.4
**IL-6**	1	1
**IL-1β**	-1.3	-1.1

*Relative mRNA expression ratios (DMSO compared to control) of the analyzed genes were normalized to hGAPDH, by qPCR.
